# Genetic deletion of S6k1 does not rescue the phenotypic deficits observed in the R6/2 mouse model of Huntington’s disease

**DOI:** 10.1038/s41598-019-52391-3

**Published:** 2019-11-06

**Authors:** Elaine E. Irvine, Loukia Katsouri, Florian Plattner, Hind Al-Qassab, Rand Al-Nackkash, Gillian P. Bates, Dominic J. Withers

**Affiliations:** 1 MRC London Institute of Medical Sciences, Imperial College London, Hammersmith Campus, London, W12 0NN UK; 2 Institute of Clinical Sciences, Faculty of Medicine, Imperial College London, Du Cane Road, London, W12 0NN UK; 3 Department of Psychiatry, University of Texas Southwestern Medical Center, Dallas, TX USA; 4 Department of Medical and Molecular Genetics, King’s College London, London, SE1 9RT UK; 5 Huntington’s Disease Centre, Department of Neurodegenerative Disease and UK Dementia Research Institute, UCL Queen Square Institute of Neurology, University College London, London, WC1N 3BG UK

**Keywords:** Genetics of the nervous system, Huntington's disease

## Abstract

Huntington’s disease (HD) is a fatal inherited autosomal dominant neurodegenerative disorder caused by an expansion in the number of CAG trinucleotide repeats in the huntingtin gene. The disease is characterized by motor, behavioural and cognitive symptoms for which at present there are no disease altering treatments. It has been shown that manipulating the mTOR (mammalian target of rapamycin) pathway using rapamycin or its analogue CCI-779 can improve the cellular and behavioural phenotypes of HD models. Ribosomal protein S6 kinase 1 (S6K1) is a major downstream signalling molecule of mTOR, and its activity is reduced by rapamycin suggesting that deregulation of S6K1 activity may be beneficial in HD. Furthermore, *S6k1* knockout mice have increased lifespan and improvement in age-related phenotypes. To evalute the potential benefit of *S6k1* loss on HD-related phenotypes, we crossed the R6/2 HD model with the long-lived *S6k1* knockout mouse line. We found that *S6k1* knockout does not ameliorate behavioural or physiological phenotypes in the R6/2 mouse model. Additionally, no improvements were seen in brain mass reduction or mutant huntingtin protein aggregate levels. Therefore, these results suggest that while a reduction in S6K1 signalling has beneficial effects on ageing it is unlikely to be a therapeutic strategy for HD patients.

## Introduction

Huntington’s disease (HD) is an inherited autosomal dominant neurodegenerative disorder that typically manifests in midlife, although childhood onset may also occur^1–4^. Symptoms begin gradually and the disease progresses to death within approximately 15–20 years of onset. HD is characterized by motor dysfunction, cognitive decline and emotional, behavioural and psychiatric disturbances, for which at present there are no effective therapies^3^. In addition, HD patients also suffer from other less well-known symptoms such as weight loss, sleep disturbances and diabetes^5–8^.

HD patients carry a mutant variant of the *HD* gene that encodes for a multi-functional scaffold protein named mutant huntingtin (mHTT). In HD patients, the *HTT* gene encodes an expanded CAG trinucleotide repeat leading to protein with abnormally long polyglutamine tracts^1,4^. Normal individuals have CAG repeat sizes of 35 or fewer, whereas HD sufferers have 36 or more and 40 CAGs is a fully penetrant mutation^9^. The abnormally long polyglutamine stretch causes the protein to misfold and accumulate in nuclear and cytoplasmic aggregates that are believed to have toxic properties leading to neuronal dysfunction and neuronal death^10^. The cerebral cortex and the striatum are especially susceptible to neuronal loss but as the disease progresses it becomes more widespread and in the latter stages of the disease neuronal death is identified in most regions of the brain^11,12^.

A number of mouse models have been generated to study the pathogenesis of HD^13–15^. The R6/2 model is one of the most commonly used and it expresses exon 1 of the human *HD* gene cloned from a HD patient; it is very well characterized and has an early onset and rapid detrimental phenotype that recapitulates many features of the human disease^16^. R6/2 mice develop a progressive deficit characterized by locomotor disturbances, weight loss, cognitive impairments and diabetes^17–22^. They also have the neuronal atrophy and intra-nuclear inclusions that are neuropathological hallmarks of clinical HD^16,23^.

A number of studies have demonstrated that inhibition of the mTOR pathway attenuated the pathological effects induced by mHTT. Rapamycin, an inhibitor of the key nutrient signal integrating protein mTORC, attenuated mHTT accumulation and cell death in cell culture models of HD, and also protected against degeneration of photoreceptor neurons in a fly overexpressing 120-CAG repeat huntingtin in the eye^24^. Furthermore, the rapamycin analogue CCI-779 improved rotarod performance and grip strength of the Ross/Borchelt HD mouse model, which has a late disease onset^24^. Everolimus, which binds with high affinity to FKB12 and thereby inhibits mTOR, decreased the phosphorylation of the mTOR kinase target protein S6 kinase and delayed the decline in motor coordination, as well as reducing the levels of soluble mHTT in the skeletal muscle^25^. Moreover, mHTT enhanced mTORC1 activity, which in turn is thought to contribute to the pathogenesis of HD. These studies therefore indicate that manipulation of the mTOR signalling pathway could be of benefit in the treatment of HD.

S6 protein kinase 1 (S6K1) is a key downstream target of mTORC1 and its activity is reduced by rapamycin via the latter’s effects on mTORC1. We have previously shown that genetic knockout of *S6k1* in mice leads to an increase in life span and resistance to age-related pathologies^26^. Previous studies in *C*. *elegans* have shown that polyglutamine aggregate accumulation and onset of toxicity in muscle is delayed in long-lived insulin/IGF-1-like pathway mutants^27^. Furthermore, a recent study showed that intercrossing heterozygous insulin like-growth factor receptor 1 (*Igf-1r)* knockout mice, which have been reported to be long-lived, with female N171-82Q HD mice delayed tremor onset in this HD model^28^. This finding suggests that the rate of progression of HD may be linked with the genetic regulation of aging. Based on this, and the studies showing that treatment with rapamycin and its analogues can ameliorate HD pathogenesis, we hypothesized that loss of *S6k1* may alleviate the symptoms observed in the R6/2 mouse model of HD.

To address this question, we generated R6/2 mice lacking *S6k1* (R6/2 × *S6k1*^−/−^) by crossing these animals with global *S6k1* knockout mice and measured body weight, locomotor activity, rotarod performance, forelimb strength, blood glucose and insulin levels, brain weight and mHTT aggregate load. We showed that genetic knockdown of *S6k1* had no beneficial effect on the levels of aggregated mHTT or on any of the behavioural or physiological deficits observed in the R6/2 mouse model, indicating that inhibition of S6K1 is unlikely to be of benefit in the treatment of HD.

## Results

In order to investigate whether deletion of *S6k1* could ameliorate the pathogenesis of HD, we used a genetic approach to delete *S6k1* in the R6/2 mouse model of HD. To generate the mice for these studies R6/2 × *S6k1*^+/−^ males were crossed with *S6k1*^+/−^ females. Offspring were born in the expected Mendelian ratios and the R6/2, R6/2 × *S6k1*^+/−^ and R6/2 × *S6k1*^−/−^ mice had well matched CAG repeat sizes (R6/2 = 256–275; R6/2 × *S6k1*^+/−^ = 258–268; R6/2 × *S6k1*^−/−^ = 254–271). Western blot analysis confirmed that S6K1 was deleted in both the *S6k1*^−/−^ and R6/2 × *S6k1*^−/−^ mice (Fig. S1).

### Deletion of *S6k1* has no beneficial effect on the body weight loss observed in the R6/2 mouse model

**Figure 1:**
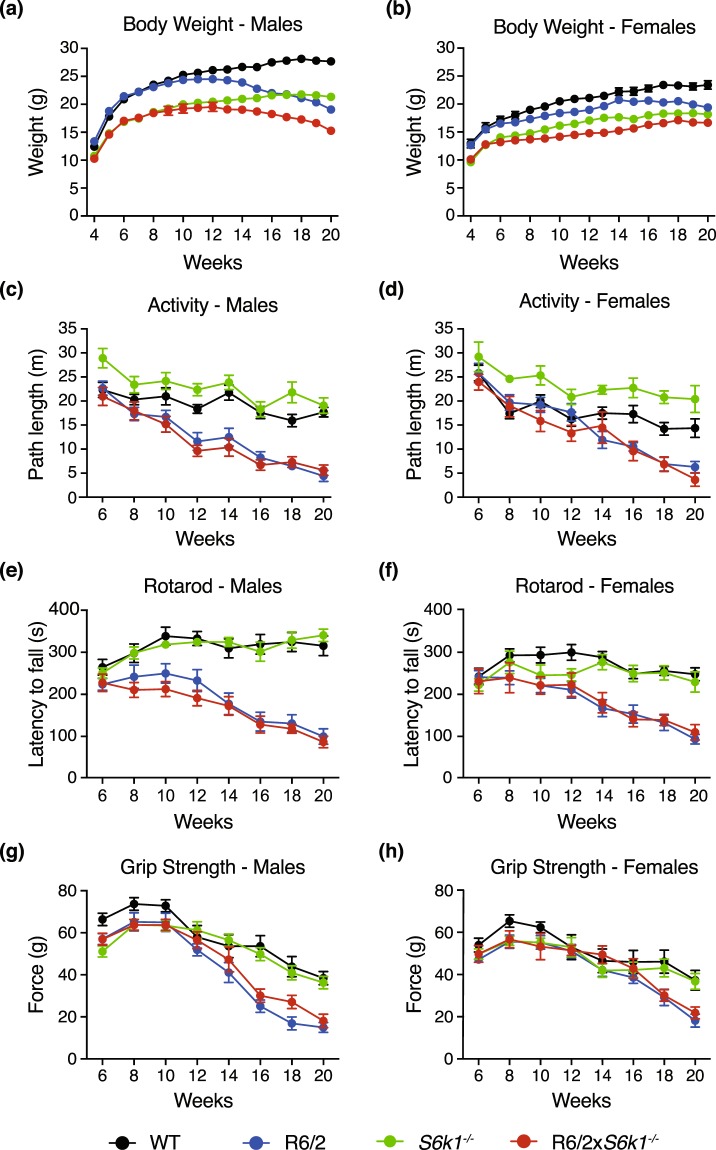
Genetic deletion of *S6k1* does not alter body weight or behavioural phenotypes of the R6/2 mouse model of HD. Body weight of male (**a**) and female (**b**) mice is shown from 4 to 20 weeks of age. The *S6k1*^−/−^ mice had reduced body weight compared to their WT littermates but deletion of *S6k1* did not affect the rate of weight loss observed in the R6/2 mice. (**c**) Male and (**d**) female mice locomotor activity was represented by path length and recorded fortnightly from 6 to 20 weeks of age. *S6k1* silencing did not improve the decrease in activity observed in the R6/2 mice. (**e**) Male and (**f**) female mice rotarod performance is presented as the average latency to fall and recorded fortnightly from 6 to 20 weeks. Genetic deletion of *S6k1* did not ameliorate the impairment in rotarod performance seen in R6/2 mice. (**g**) Male and (**h**) female mice average grip strength is presented fortnightly from 6 to 20 weeks. *S6k1* deletion did not significantly improve the impairment in average grip strength observed in the R6/2 mice. For all studies, WT, males n = 9 and females n = 9, R6/2, males n = 9 and females n = 11, *S6k1*^−/−^, males n = 11 and females n = 8, R6/2 × *S6k1*^−/−^, males n = 8 and females n = 7. Data were analysed by general linear model (GLM) with repeated measures and are presented as mean ± SEM.

R6/2 mice maintain normal body weight during the first part of their lives, but gradually lose weight after the onset of the disease, a phenotype that resembles the low BMI reported for HD patients^5^. In this study, male and female mice were weighed weekly from 4 to 20 weeks of age (Fig. 1a,b; Table 1). Consistent with previous findings, both male and female R6/2 mice weighed significantly less overall than their control littermates (males: F_1,33_ = 27.356, P < 0.001; females: F_1,31_ = 19.877, P < 0.001), and had significant weight loss over time (males: F_16,528_ = 52.465, P < 0.001; females: F_16,496_ = 9.954, P < 0.001). As anticipated, *S6k1*^−/−^ mice were dwarves and so were also significantly smaller than their WT littermates (males: F_1,33_ = 119.521, P < 0.001; females: F_1,31_ = 113.564, P < 0.001). Genetic knockout of *S6k1* in R6/2 mice did not attenuate the rate of weight loss as the R6/2 × *S6k1*^−/−^ mice began to progressively lose weight at a similar age, and rate, as the R6/2 mice (males: F_16,528_ = 3.185, P = 0.036; females: F_16,496_ = 2.976, P = 0.015). Therefore, *S6k1* deletion in the R6/2 mouse model did not improve the weight loss phenotype.

**Table 1:** Summary of statistical values for the physiological and behavioural studies.

Factor	Males				Females			
	Body Weight	Activity - Path Length	Rotarod	Grip Strength	Body Weight	Activity - Path Length	Rotarod	Grip Strength
**Within-Subject Effects**								
Age	*P* < *0*.*001*	*P* < *0*.*001*	*P* < *0*.*001*	*P* < *0*.*001*	*P* < *0*.*001*	*P* < *0*.*001*	*P* < *0*.*001*	*P* < *0*.*001*
Age*R6/2	*P* < *0*.*001*	*P* < *0*.*001*	*P* < *0*.*001*	*P* < *0*.*001*	*P* < *0*.*001*	*P* < *0*.*001*	*P* < *0*.*001*	*P* < *0*.*005*
Age*S6k1^−/−^	*P* = *0*.*008*	P = 0.281	P = 0.418	P = 0.065	*P* < *0*.*001*	P = 0.557	P = 0.792	P = 0.728
Age*R6/2*S6k1^−/−^	*P = 0.036*	P = 0.274	P = 0.671	P = 0.746	*P = 0.015*	P = 0.707	P = 0.625	P = 0.858
**Between-Subject Effects**								
R6/2	*P* < *0*.*001*	*P* < *0*.*001*	*P* < *0*.*001*	*P* < *0*.*001*	*P* < *0*.*001*	*P* < *0*.*001*	*P* < *0*.*001*	*P = 0*.*018*
*S6k1* ^−/−^	*P* < *0*.*001*	P = 0.271	P = 0.519	P = 0.717	*P* < *0*.*001*	P = 0.080	P = 0.503	P = 0.705
R6/2*S6k1^−/−^	P = 0.388	P = 0.084	P = 0.593	*P = 0*.*046*	P = 0.518	*P* < *0*.*005*	P = 0.366	P = 0.189

The numbers displayed indicate the P values for each of the parameters analysed, genotype (R6/2), treatment (*S6k1*^−*/*−^) and age. Data were analysed by general linear model (GLM) with repeated measures. All significant effects are in italics.

### Motor function and muscle strength are not improved in the R6/2 mouse model with *S6k1* deletion

Exploratory behaviour was assessed fortnightly from 6 to 20 weeks of age using the open field test (Fig. 1c,d; Table 1). As previously shown, male and female R6/2 mice were significantly less active than their control littermates (males: F_1,33_ = 59.665, P < 0.001; females: F_1,31_ = 34.881, P < 0.001), and became more hypoactive with age (males: F_7,231_ = 10.707, P < 0.001; females: F_7,217_ = 7.542, P < 0.001). The *S6k1*^−/−^ mice showed a trend for being hyperactive, but this did not reach significance for either gender (males: F_1,33_ = 1.266, P = 0.271; females: F_1,31_ = 3.265, P = 0.080). The loss of *S6k1* had no influence on the R6/2 hypoactivity phenotype as the R6/2 × *S6k1*^−/−^ showed a decline in activity at a similar rate to that seen in the R6/2 mice (males: F_7,231_ = 1.282, P = 0.274; females: F_7,217_ = 0.593, P = 0.707). We also showed that heterozygote deletion of *S6k1* did not affect the hypoactivity observed in the R6/2 mice as R6/2 × *S6k1*^+/−^ mice showed a similar decline in overall activity as both the R6/2 and R6/2 × *S6k1*^−/−^ mice (F_2,18_ = 0.389, P = 0.683) (Fig. S2a)

Fore- and hind-limb motor coordination and balance, which have been shown to be impaired in the R6/2 mice as the disease progresses, were assessed fortnightly from 6 to 20 weeks of age using the rotarod (Fig. 1e,f; Table 1). Consistent with previous reports both male and female R6/2 mice had a significant decline in rotarod performance (males: F_1,33_ = 78.827, P < 0.001; females: F_1,31_ = 32.232, P < 0.001) that worsened with age (males: F_7,231_ = 28.297, P < 0.001; females: F_7,217_ = 9.681, P < 0.001). The performance of the *S6k1*^−/−^ mice was not different to their control littermates (males: F_1,33_ = 0.425, P = 0.519; females: F_1,31_ = 0.458, P = 0.503) and did not change with age (males: F_7,231_ = 0.995, P = 0.418; females: F_7,217_ = 0.408, P = 0.792). Loss of *S6k1* did not modify the performance of R6/2 mice overall (males: F_1,33_ = 0.291, P = 0.593; females: F_1,31_ = 0.841, P = 0.366) or with age (males: F_7,231_ = 0.617, P = 0.671; females: F_7,217_ = 0.642, P = 0.625). Furthermore, we showed that heterozygote deletion of *S6k1* did not affect the reduced performance observed in the R6/2 mice as R6/2 × *S6k1*^+/−^ mice showed a similar decline in overall performance as both the R6/2 and R6/2 × *S6k1*^−/−^ mice (F_2,18_ = 0.305, P = 0.741) (Fig. S2b). These results show that genetic ablation of *S6k1* does not improve the motor coordination and balance of the R6/2 mice.

Forelimb grip strength was measured fortnightly from 6 to 20 weeks of age (Fig. 1g,h; Table 1). The overall grip strength of the R6/2 mice was significantly reduced compared to their control littermates (males: F_1,33_ = 35.683, P < 0.001; females: F_1,31_ = 6.261, P = 0.018), and deteriorated over time (males: F_7,231_ = 7.600, P < 0.001; females: F_7,217_ = 4.036, P < 0.005). The *S6k1*^−/−^ mice had similar forelimb grip strength to their WT littermates (males: F_1,33_ = 0.134, P = 0.717; females: F_1,31_ = 0.146, P = 0.705), that did not differ with age (males: F_7,231_ = 2.214, P = 0.065; females: F_7,217_ = 0.519, P = 0.728). Loss of *S6k1* led to a small but significant improvement in forelimb grip strength of male R6/2 mice overall (F_1,33_ = 4.295, P = 0.046), although no difference was seen with age (F_7,231_ = 0.505, P = 0.746). However, female R6/2 × *S6k1*^−/−^ mice showed no improvement in grip strength (F_1,33_ = 1.803, P = 0.189) or with age (F_7,231_ = 0.338, P = 0.858). Additionally, we showed that heterozygote deletion of *S6k1* did not affect the reduced performance observed in the R6/2 mice as R6/2 × *S6k1*^+/−^ mice showed a similar decline in performance over time as both the R6/2 and R6/2 × *S6k1*^−/−^ mice (F_2,18_ = 0.389, P = 0.683) (Fig. S2c). Therefore, genetic depletion of *S6k1* may have a small improvement in forelimb muscle strength of male R6/2 mice but does not affect females.

### *S6k1* deletion did not attenuate the increased fed and fasted glucose and insulin levels observed in the R6/2 mice

**Figure 2:**
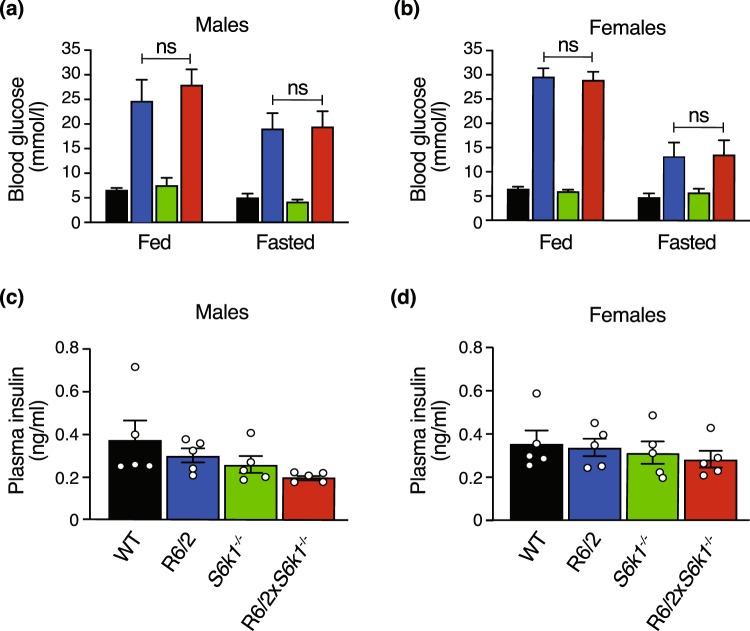
Genetic deletion of *S6k1* does not alter fed and fasted blood glucose and insulin levels in the R6/2 mouse model of HD. (**a**) Male and (**b)** female mice fed and fasted blood glucose levels were measured at 20 weeks of age. Deletion of *S6k1* did not reduce the elevated blood glucose observed in R6/2 mice in the fed or fasted state. Males n = 6–8/genotype and females n = 7–10/genotype. ns = non-significant and P > 0.999 for all. (**c**) Male and (**d**) female R6/2 and R6/2 × *S6k1*^−/−^ insulin levels were unaltered. Males and females n = 5/genotype. Data were analysed with GLM univariate with Bonferroni correction and are presented as mean ± SEM.

HD patients have been shown to have alterations in glucose metabolism and diabetes, which are recapitulated in the R6/2 mouse model^19,20^. We measured fed and fasted blood glucose levels in 20 week old male and female mice (Fig. 2a,b). Consistently, we show here that both male and female R6/2 mice had increased fed (males: F_1,23_ = 57.580, P < 0.001; females: F_1,32_ = 319.991, P < 0.001) and fasted (males: F_1,23_ = 47.495, P < 0.001; females: F_1,32_ = 12.152, P < 0.001) blood glucose levels at 20 weeks of age. The *S6k1*^−/−^ mice had normal fed (males: F_1,23_ = 0.694, P = 0.413; females: F_1,32_ = 0.207, P = 0.652) and fasted (males: F_1,23_ = 0.011, P = 0.916; F_1,32_ = 0.087, P = 0.770) blood glucose. Ablation of *S6k1* did not attenuate the increase in fed (males: P > 0.999; females > 0.981) and fasted (males and females: P > 0.999) blood glucose levels observed in R6/2 mice. Furthermore, we showed that R6/2 × *S6k1*^+/−^ mice had similar fed (H(2) = 0.116, P = 0.944) and fasted (H(2) = 0.401, P = 0.818) blood glucose levels as R6/2 and R6/2 × *S6k1*^−/−^ mice showing that heterozygote deletion of *S6k1* did not improve blood glucose levels (Fig. S2d).

Insulin levels were normal in R6/2 (males: F_1,16_ = 1.628, P = 0.220; females: F_1,16_ = 0.251, P = 0.623) and *S6k1*^−/−^ (males: F_1,16_ = 4.372, P = 0.053; females: F_1,16_ = 0.251, P = 0.623) mice at 20 weeks of age, and deletion of *S6k1* had no effect on the insulin levels in R6/2 mice (males: P = 0.533 and females: P = 0.858) (Fig. 2c,d).

### Neuropathological characteristics observed in the R6/2 mouse model were unaffected by *S6k1* deletion

**Figure 3:**
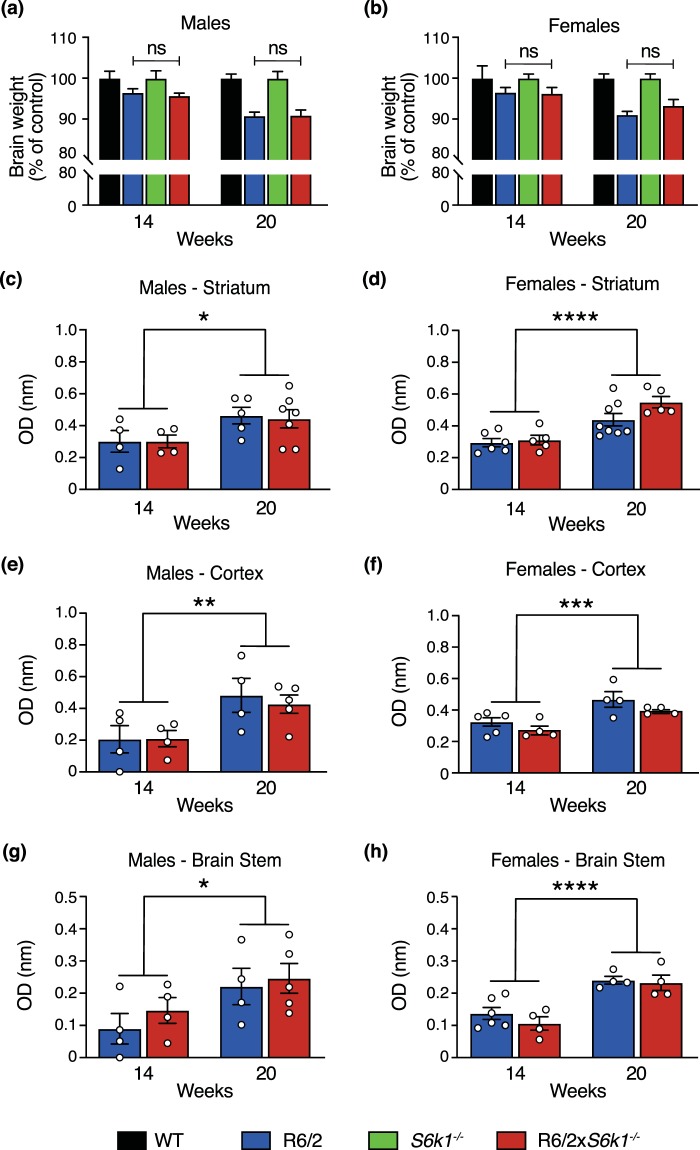
Deletion of *S6k1* does not reverse the brain weight reduction or the mHTT aggregation observed in R6/2 mice. (**a**) Male and (**b**) female mouse brain weight was measured at 14 and 20 weeks. As the *S6k1*^−/−^ mice have smaller brains compared to WT mice data are represented as % of appropriate control, R6/2 to WT and R6/2 × *S6k1*^−/−^ to *S6k1*^−/−^. Deletion of *S6k1* did not modify the brain weight loss in R6/2 mice as the % reduction in brain weight was equivalent for R6/2 and R6/2 × *S6k1*^−/−^ mice. For 14 weeks, males n = 4–9/genotype and females n = 4–5/genotype and for 20 weeks, males n = 12–14/genotype and females n = 14/genotype. Data were analysed by GLM univariate with Bonferroni correction. ns = non-significant and P > 0.999 for all. (**c**–**f**) The Seprion ligand based ELISA assay was used to quantify mHTT aggregation in the striatum, cortex and brain stem of (a, c and e) male and (b, d and f) female R6/2 and R6/2 × *S6k1*^−/−^ mice. Aggregate levels increase with age but are not modified by *S6k1* deletion. For 14 weeks, males n = 4/genotype and females n = 4–6/genotype and for 20 weeks, males n = 4–7/genotype and females n = 4–8/genotype. Data were analysed with GLM univariate with Bonferroni correction and are presented as mean ± SEM. *P <0.05, **P < 0.01, ***P < 0.005 and ****P < 0.001.

Neuropathological features of R6/2 mice include decreased brain size^23^. In this study mice were sacrificed at 14 and 20 weeks of age and brains were weighed to the nearest 0.001 g (Figs 3a,b, S3a,b). As expected we found that the R6/2 mice had decreased brain weight compared to their control littermates (males: F_1,68_ = 33.001, P < 0.001; females: F_1,65_ = 26.059, P < 0.001), which reduced with age (males: F_1,68_ = 5.635, P = 0.020; females: F_1,65_ = 3.811 P = 0.055). The *S6k1*^−/−^ mice also had significantly reduced brain weight compared to the WT controls (males: F_1,68_ = 59.071, P < 0.001; females: F_1,65_ = 70.495, P < 0.001) although as expected this did not alter with age (males: F_1,68_ = 1.509, P = 0.224; females: F_1,65_ = 3.811, P = 0.064). Similar to the behavioural phenotypes, knockout of *S6k1* did not attenuate the brain mass reduction seen in the R6/2 mice (males: F_1,68_ = 0.067, P = 0.796; females: F_1,65_ = 0.378, P = 0.541). A similar reduction in brain mass was also observed in the R6/2 × *S6k1*^+/−^ mice, showing that heterozygote deletion of *S6k1* did not reverse this reduction (F_2,30_ = 1.130, P = 0.337) (Fig. S2e).

Aggregation of mHTT is a classic feature of HD neuropathology^9,10^. Inclusions of mHTT have also been observed throughout the brain and in many tissues of the R6/2 model^29^. We assessed whether deletion of *S6k1* decreased the aggregate load that was observed in the R6/2 mice using a highly sensitive Seprion ELISA^30^. This assay detects aggregated mHTT protein in tissue lysates by immunoprobing with an antibody raised against the N-terminal exon 1 HTT protein^30^. We quantified the aggregate load in the striatum, cortex and brain stem of R6/2 and R6/2 × *S6k1*^−/−^ mice at 14 and 20 weeks of age (Fig. 3c–h). We found that in both male and female mice the amount of aggregated mHTT in the striatum (males: F_1,16 = _6.608, P = 0.021; females: F_1,20_ = 28.690, P < 0.001) cortex (males: F_1,13_ = 10.276, P = 0.007; females: F_1,14_ = 17.339, P = 0.001) and brain stem (males: F_1,13_ = 5.753, P = 0.032; females: F_1,14_ = 33.282, P < 0.001) increased from 14 to 20 weeks of age. However, no difference was observed between the genotypes at either time point for the striatum (males: F_1,16_ = 0.029, P = 0.867; females: F_1,20_ = 3.132, P = 0.092), cortex (males: F_1,13_ = 0.112, P = 0.743; females: F_1,14_ = 3.569, P = 0.080) and brain stem (males: F_1,13_ = 0.731, P = 0.408; females: F_1,14_ = 0.961, P = 0.344). Our data show that genetic ablation of *S6k1* does not alter mHTT aggregate load in the striatum, cortex and brainstem of R6/2 mice. We also showed that heterozygote deletion of *S6k1* did not alter mHTT aggregate load in the striatum as levels in R6/2 × *S6k1*^+/−^ mice were similar to those observed in R6/2 and R6/2 × *S6k1*^−/−^ mice (F_2,14_ = 1.734, P = 0.212) (Fig. S2f).

## Discussion

Previous studies, in both cells and model organisms, have suggested that attenuation of mTORC1 signalling may provide therapeutic benefit in the treatment of HD. For example, rapamycin treatment of *Drosophila* expressing mHTT in their photoreceptors reduced neurodegeneration in the eye^24^. Likewise, in the Ross/Borchelt mouse model of HD, treatment with the rapamycin ester CCI-779 improved a range of neurological parameters including rotarod performance and grip strength, and reduced HTT aggregates in the brain^24^. The underlying mechanisms for this beneficial effect have been suggested to involve the induction of autophagy upon inhibition of mTOR and subsequent clearance of polyglutamine aggregates. Consistent with this hypothesis it has also been shown that HTT itself promotes mTORC1 signalling and increased mTOR activity in the striatum of N171-82QHD mice exacerbates neurological phenotypes^31^. Thus, increased activity of the mTORC1 may in turn contribute to HD pathogenesis, providing further rationale for therapeutically targeting this pathway.

S6K1 is a major downstream effector of mTORC1 signalling. A broad range of studies have implicated S6K1 in a number of cellular processes including protein translation, ribosomal biogenesis, mRNA splicing, transcription, cytoskeletal organization, regulation of cell signalling intermediates and cell proliferation^32–35^. At an organismal level S6K1 signalling has been shown to control growth and metabolism including glucose homeostasis and insulin sensitivity. Importantly, S6K1 has been shown to control the ageing process as mice lacking *S6k1* globally are long-lived and resistant to the diseases of ageing^26^. Moreover, tissue-specific loss of *S6k1* reduces the accelerated pathology and physiological decline associated with loss of lamin A^36^. Furthermore, and of relevance to HD, genetic reduction of *S6k1* has been shown to reduce neurological defects and neuropathology in the triple transgenic mouse model of Alzheimer’s disease^37^. Taken together these findings suggest a connection between S6K1, neurodegeneration and ageing, and motivated our studies to investigate whether loss of *S6k1* may ameliorate the disease course in the R6/2 mouse model of HD.

In our studies, we found that global deletion of *S6k1* did not attenuate the features of HD in the R6/2 mouse model. Abrogation of *S6k1* had no effects on aggregated mHTT load in the striatum, cortex or brainstem. This was reflected in the failure to see an improvement in a range of behavioural parameters such as locomotor activity, rotarod performance and grip strength. This lack of benefit was seen in both male and female mice. *S6k1*^−/−^ mice display growth retardation which in part complicated the interpretation of body weight profiles in the R6/2 × *S6k1*^−/−^ intercross but again for this parameter there was no apparent benefit in male animals.

R6/2 mice display abnormalities of glucose homeostasis with elevated fasting blood glucose levels, reduced insulin levels and impaired glucose tolerance^19,20^. *S6k1*^−/−^ mice have increased insulin sensitivity and improved glucose homeostasis under conditions of nutrient excess and in old age^26,38^. It therefore might have been anticipated that reduction of *S6k1* might show improvement in glucose handling. However, fed and fasting blood glucose levels and glucose tolerance showed no improvements in both R6/2 × *S6k1*^+/−^ and R6/2 × *S6k1*^−/−^ mice. Therefore, taken together it is clear that global genetic reduction of *S6k1* does not improve the neurological features of R6/2 mice or result in any metabolic benefits.

There are a number of potential explanations for the failure to observe improvements in R6/2 HD phenotypes. The beneficial effects of rapamycin-induced mTORC1 inhibition may be due to the more general effects upon the pathway than the specific deletion of just one of its downstream effectors. It appears that the induction of autophagy by rapamycin is key to its effects upon HTT aggregate clearance^39^. While S6K1 has many molecular targets and cellular effects the induction of autophagy is not a prominent and consistent finding with deletion of *S6k1*. Furthermore, rapamycin inhibits the activation both S6K1 and S6K2 and the beneficial effects may require suppression of both these kinases. Therefore, it is conceivable that simply deleting *S6k1* alone does not capture the underlying signalling and cellular events required to see a therapeutic benefit. Consistent with this concept, deletion of *Igf1r*, an upstream activator of S6K1 signalling, had some benefits in N171-82Q HD mice, but this manipulation was not associated with a reduction in the phosphorylation p70S6k, suggesting that suppression of S6K1 activity was not mediating these effects^28^. It should also be noted that this study demonstrates both beneficial and detrimental effects on HD pathology indicating complexity in the signalling pathways. The beneficial effects of *S6k1* deletion upon AD pathology probably reflects the different aetiopathophysiology of HD and AD although both are aggregation-associated neurological diseases.

An additional reason for the lack of a beneficial effect could be the choice of HD model used. In the original description of the beneficial effects of rapamycin on HD, the Ross/Borchelt mouse model was used^24,40^, which has a slightly longer life expectancy and slight delay in some behavioural features compared to the R6/2 model. Although our manipulation of *S6k1* occurred in the germ-line the more progressive R6/2 phenotype could have overcome any potential beneficial effects of the loss of *S6k1*. Indeed, treatment of R6/2 mice with everolimus, which acts with a very similar mechanism to rapamycin but with more mTORC1 selectivity, has subsequently been reported to not reduce brain HTT aggregates or have beneficial neuroprotective effects but did alter skeletal muscle HTT load. Interestingly, in this study everolimus suppressed brain rpS6 phosphorylation suggesting inhibition of S6K1 activity but did not induce autophagy^25^.

In summary while abrogation of S6K1 signalling has beneficial effects on ageing and age-related disease including attenuation of the pathology seen in AD, this therapeutic effect does not extend to the amelioration of the HD phenotype in the R6/2 mouse model of HD.

## Materials and Methods

### Animals

B6.Tg(HDexon1)62Gpb/240JChdi (R6/2) transgenic breeding males, transgenic for the 5′ end of the human m*HTT* gene with an expanded CAG repeat^17^, were obtained from the CHDI Foundation stock at Jackson Laboratories (CHDI Number: CHDI-81001006; Jax Stock Number: 371097; Bar Harbor, ME). These R6/2 mice were crossed with null B6.*Rps6kb1*^*tm1Gtho*^ mice (*S6k1*^−/−^ mice)^26^, to generate R6/2 mice that were heterozygous for B6.*Rps6kb1*^*tm1Gtho*^ (R6/2 × *S6k1*^+/−^ mice). R6/2 × *S6k1*^+/−^ males from this cross were then bred with *S6k1*^+/−^ females to generate the desired genotypes, WT, R6/2, *S6k1*^−/−^ and B6.Tg(HDexon1)62Gpb/240JCh × B6.*Rps6kb1*^*tm1Gtho*^ (R6/2 × *S6k1*^−/−^) mice. From this cross a small number of R6/2 × *S6k1*^+/−^ females were kept for analysis. Mice were housed in groups of three to six of mixed genotype and maintained on a 12 h light/dark cycle with food and water ad libitum. All behavioural experiments were conducted between 9 am and 1 pm. Where possible experimenters were blinded to genotype of both live animals and tissue and blood samples. All experiments were undertaken in accordance with the UK Animals (Scientific Procedures) Act 1986 as well as being approved by Imperial College Animal Welfare and Ethical Review Body and by the UK Home Office. Findings and experiments described in this paper were designed and reported following the Animal Research: Reporting of *In Vivo* Experiments (ARRIVE) guidelines^41^.

### Genotyping and CAG repeat sizing

WT, R6/2, *S6k1*^−/−^ and R6/2 × *S6k1*^−/−^ mice were identified by PCR with DNA obtained from tail biopsies on postnatal day 21, the day of weaning. To genotype for R6/2, forward primer 33727 (5′-CGCAGGCTAGGGCTGTCAATCATGCT-3′) and reverse primer 32252 (5′-TCATCAGCTTTTCCAGGGTCGCCAT-3′) were used. The amplified transgene product was 272 bp. For S6K1, three primers were used, forward primer F377 (5′-CCCATCTTTACTGAAGGAGCTACT), reverse primer R377 (5′- AGGCTGGACTCAAACTCATAGAGA-3′) and neo100 (5′-GCCTTCTTGACGAGTTCTTCTGA-3′). The amplified WT product was 375 bp and the *S6k1* KO product was 175 bp. The CAG repeat size of the R6/2 mice and double mutants was determined by a FAM labelled forward primer (5′-GAGTCCCTCAAGTCCTTCCAGCA-3′) and reverse primer (5′-GCCCAAACTCACGGTCGGT-3′). The FAM-tagged PCR product was analysed using an ABI3730 sequencer and analysed with the GeneMarker software (SoftGenetics).

### Western blot analysis

Brains were removed and homogenized in lysis buffer (50 mM Tris pH 7.4, 150 mM NaCl, 1 mM EDTA, 0.1% w/v Triton X-100) supplemented with Roche Complete protease inhibitor cocktail, using a motorised pellet mixer with autoclaved polypropylene pestles. Twenty μg of total protein homogenates were resolved in 10% SDS-PAGE gels, transferred to PVDF membranes using a wet transfer method (BioRad) and incubated for 1 h with anti-p70 S6 Kinase antibody (49D7) (Rabbit mAb, 1:3000, #2708 S; Cell Signalling) in blocking solution (5% milk in TBS-Tween 0.1%). Tubulin was used as loading control (1:10,000, #T5293 Sigma). Detection was performed using enhanced chemiluminescence (Luminata Crescento, Millipore) and film exposure (Amersham Biosciences).

### Body weight

Body weight was measured at the same time of the day each week from 4 weeks of age until 20 weeks using a Sartorius BP610 balance. The same cohort of mice was used for the body weight curves and all the behavioural tests. At the start of the study the n numbers for the males was: WT, n = 9, R6/2, n = 9, *S6k1*^−/−^, n = 11 and R6/2 × *S6k1*^−/−^. n = 9, and for the females was: WT, n = 9, R6/2, n = 11, *S6k1*^−/−^, n = 8 and R6/2 × *S6k1*^−/−^. n = 9. One male and 2 female R6/2 × *S6k1*^−/−^ mice had to be culled before termination of the study due to their weight loss reaching that of the limits of the Home Office PPL.

### Open field

Locomotor activity was assessed using the HVS Image tracking software (HVS Image 2100, Hampton, UK). Mice were individually placed in a wooden grey arena (45 × 45 × 30 cm) that had a base covered in sawdust. Each mouse was released into a corner of the box and was allowed to explore for 5 min. The tracking system recorded distance travelled. Testing began at 6 weeks of age and mice were tested every 2 weeks until they reached 20 weeks of age.

### Rotarod

The rotarod apparatus (Ugo Basile, Italy) was used to measure fore- and hind-limb motor coordination, balance and strength. Mice performed three trials per day with an inter-trial interval of 1 h for three consecutive days. The rod accelerated from 5 to 60 rpm over a period of 570 s and the latency to fall was recorded. For the subsequent analysis the mean latency was taken for the 9 trials and plotted as a value for the week. Testing began at 6 weeks of age and mice were tested every 2 weeks until they reached 20 weeks of age.

### Grip strength

A grip strength meter was used to measure forelimb strength (Linton Instruments, UK). To measure grip strength the mouse was swung gently by the tail so that its forelimbs contacted the grip bar. The mouse instinctively gripped the bar and was then pulled horizontally backwards, exerting a tension until the mouse released the bar. The maximum load was recorded by the grip strength meter, which was then returned to zero before the next test. The mouse was placed back in its home cage for a minute to rest before the next test. Each mouse performed five consecutive tests, and the three highest scores were used for statistical analysis. This protocol is preferred because lower scores are often due to the mouse failing to grip the bar effectively, rather than reflecting muscular strength. Testing began at 6 weeks of age and mice were tested every 2 weeks until they reached 20 weeks of age. All scores were normalized to body weight.

### Metabolic analysis

Fed and fasted blood samples were collected from mice via tail vein or trunk bleeds using a capillary blood collection system (Sarstedt, Nümbrecht) and blood glucose was measured using a Contour glucometer (Bayer Corp). Fasted blood samples were collected after a 16 h overnight fast. Plasma insulin levels were analysed using a mouse insulin ELISA (Crystalchem Inc.).

### Seprion ligand ELISA

Brains from all genotypes were sub-dissected into specific brain regions, snap-froze in liquid nitrogen and then stored at −80 °C until required. A 2.5% lysate (w/v) was prepared in ice-cold RIPA buffer (50 mM Tris–HC pH 8.0; 120 mM NaCl; 1% Igepal; 3.125% sodium deoxycholate; 0.01% SDS; 1 mM β-mercaptoethanol; 1 mM PMSF; 1 mM DTT; protease inhibitor cocktail; Roche) by ribolysing for 30 s in Lysing matrix tubes (Lysing matrix D; MP Biomedicals). Samples were stored on ice for 5 min and used immediately. Homogenate (15 ml) was mixed with 3 ml 10% SDS, diluted to 80 ml with water, and then made up to 100 ml with 5x capture buffer (Microsens Biotechnologies). For detection of aggregated protein, lysate was transferred to the well of a Seprion ligand-coated ELISA plate, and incubated with shaking for 1 h at room temperature (RT). After removal of the lysate, the well was washed 5 times in PBS-T (PBS; 0.1% Tween) and 100 ml S830 primary anti- body (diluted 1:2000 in conjugate buffer (150 mM NaCl; 4% BSA (98% electrophoretic grade); 1% non-fat dried milk; 0.1% Tween 20 in PBS) was added and incubated with shaking for 1 h at RT. After five washes with PBS-T, 100 ml horseradish peroxidase-conjugated rabbit anti-goat secondary antibody (DAKO) (1:2000 in conjugation buffer) was added and incubated with shaking for 45 min at RT. After washing five times with PBS-T, 100 ml of TMB substrate (SerTec) at RT was added and incubated in the dark (wrapped in foil) at RT for 30 min. Reactions were terminated by the addition of 100 ml 0.5 M HCl and the absorption at 450 nm was measured using a plate reader (BioRad). For analysis, the WT mice absorption values were subtracted from those of the R6/2, R6/2 × *S6k1*^+/−^ and R6/2 × *S6k1*^−/−^ mice.

### Statistical analysis

Statistical analyses were performed using SPSS (IBM) or Prism 7 (GraphPad). For all longitudinal physiological and behavioural tests to determine the effect of either genotype or treatment over the course of the study General Linear Model repeated measures ANOVA was used with genotype (R6/2), treatment (*S6k1*^−/−^) and age as within-subject factors, with the Greenhouse–Geisser correction for non-sphericity. For all other studies, General Linear Model univariate and ANOVA analysis with Bonferroni correction, or the Kruskall Wallis test if data were not normally distributed. Normality of distribution was assessed using the Shapiro-Wilk test. Data are expressed as mean ± SEM and statistical significance is defined as p < 0.05.

## Supplementary information

Supplementary Information

## Data Availability

The datasets generated during and/or analysed during the current study are available from the corresponding author on reasonable request.
