# Supplementary material for: Genetic deletion of S6k1 does not rescue the phenotypic deficits observed in the R6/2 mouse model of Huntington’s disease
Source: Sci Rep. 2019 Nov 6;9:16133. doi: 10.1038/s41598-019-52391-3 (PMC6834565; doi:10.1038/s41598-019-52391-3)
Supplement: Supplementary file 1 — Supplementary Information [file 41598_2019_52391_MOESM1_ESM.docx]

Supplemental Information

**Genetic deletion of S6k1 does not rescue the phenotypic deficits observed in the R6/2 mouse model of Huntington’s disease**

Elaine E. Irvine, Loukia Katsouri, Florian Plattner, Hind Al-Qassab, Rand Al-Nackkash, Gillian P. Bates and Dominic J. Withers

**Supplementary Figure 1: Expression of S6K1 is abolished in *S6k1^-/-^* and R6/2x*S6k1^-/-^* mouse brain.** (a) Representative western blot analysis of S6K1 expression in whole brain of WT, *S6k1^-/-^*, R6/2 and R6/2x*S6k1^-/-^* mice using anti-p70 S6 kinase antibody (49D7) (Rabbit mAb, 1:3000, #2708S; Cell Signalling^1^). Tubulin was used as loading control (1:10,000, #T5293 Sigma^2^). (b and c) Scanned raw images for the western blot images shown in (a). The same nitrocellulose membrane was used for p70 S6 Kinase (b) and for tubulin (c). The faint band seen in lane 2 of the *S6k1^-/-^* mouse is due to a slight overspill from lane 1 when loading the gel (a and b).

**
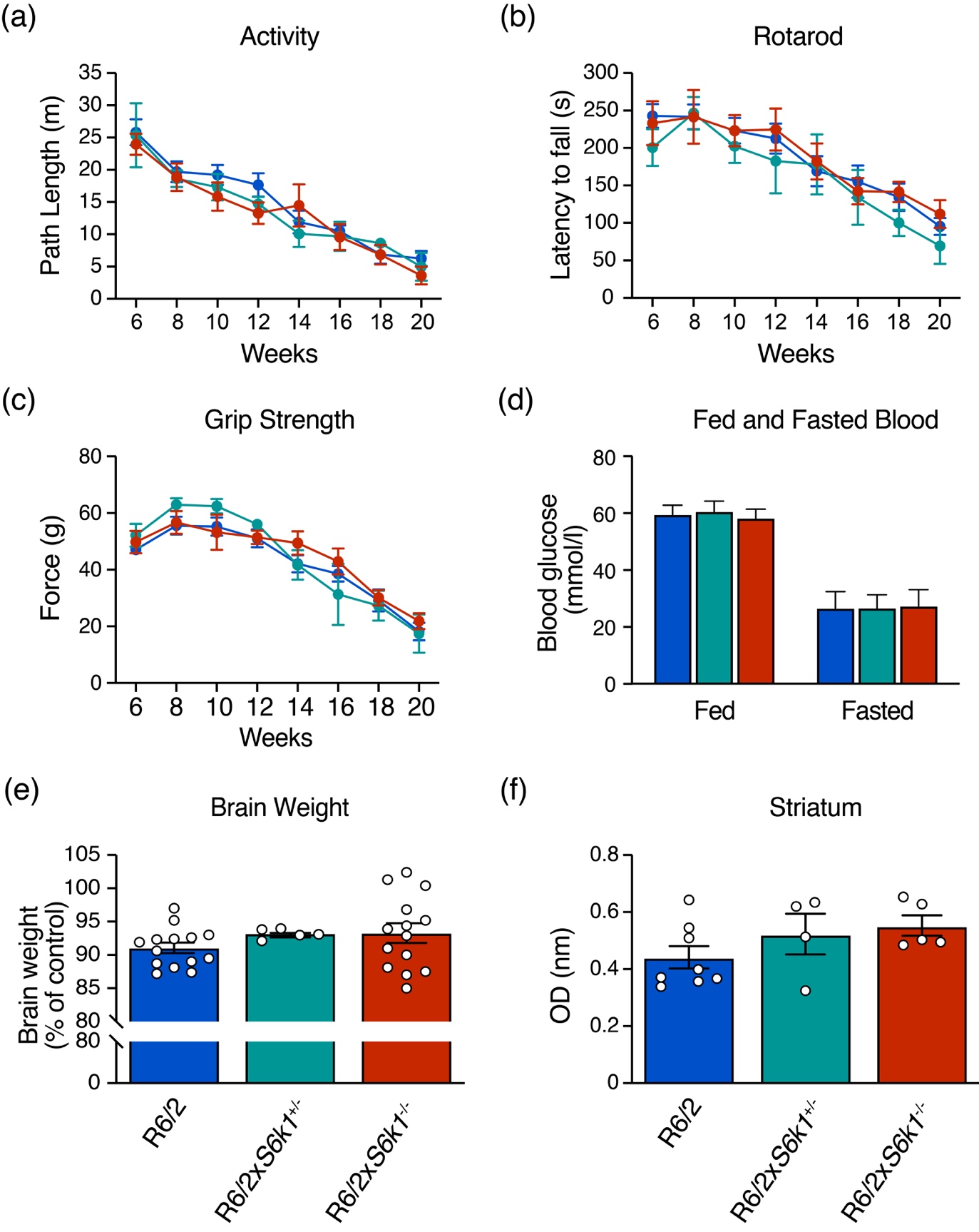
**

**Supplementary Figure 2: No change in behaviour, blood glucose, brain weight and striatal aggregate load between female R6/2, R6/2x*S6k1^+/-^* and R6/2xS6k1^-/-^ mice.** No difference in locomotor activity (a), rotarod performance (b) and grip strength (c) was observed between the R6/2, R6/2x*S6k1^+/-^* and R6/2x*S6k1^-/-^* mice. For all behavioural studies R6/2, n = 11, R6/2x*S6k1^+/^*,*^-^* n = 3, R6/2x*S6k1^-/-^*, n = 7. (d) Fed and fasted blood glucose levels were no different between the R6/2, R6/2x*S6k1^+/-^* and R6/2xS6k1^-/-^ mice. R6/2, n = 10, R6/2x*S6k1^+/^*,*^-^* n = 5, R6/2x*S6k1^-/-^*, n = 10. (e) No difference in brain weight was observed between the R6/2, R6/2x*S6k1^+/-^* and R6/2xS6k1^-/-^ mice. R6/2, n = 14, R6/2x*S6k1^+/^*,*^-^* n = 5, R6/2x*S6k1^-/-^*, n = 14. (f) Striatal mutant huntingtin aggregate levels were not different between the R6/2, R6/2x*S6k1^+/-^* and R6/2xS6k1^-/-^ mice. R6/2, n = 8, R6/2x*S6k1^+/^*,*^-^* n = 4, R6/2x*S6k1^-/-^*, n = 5. Data were analysed by general linear model (GLM) with repeated measures, ANOVA and Kruskal Wallis tests. Data are presented as mean ± SEM.

**
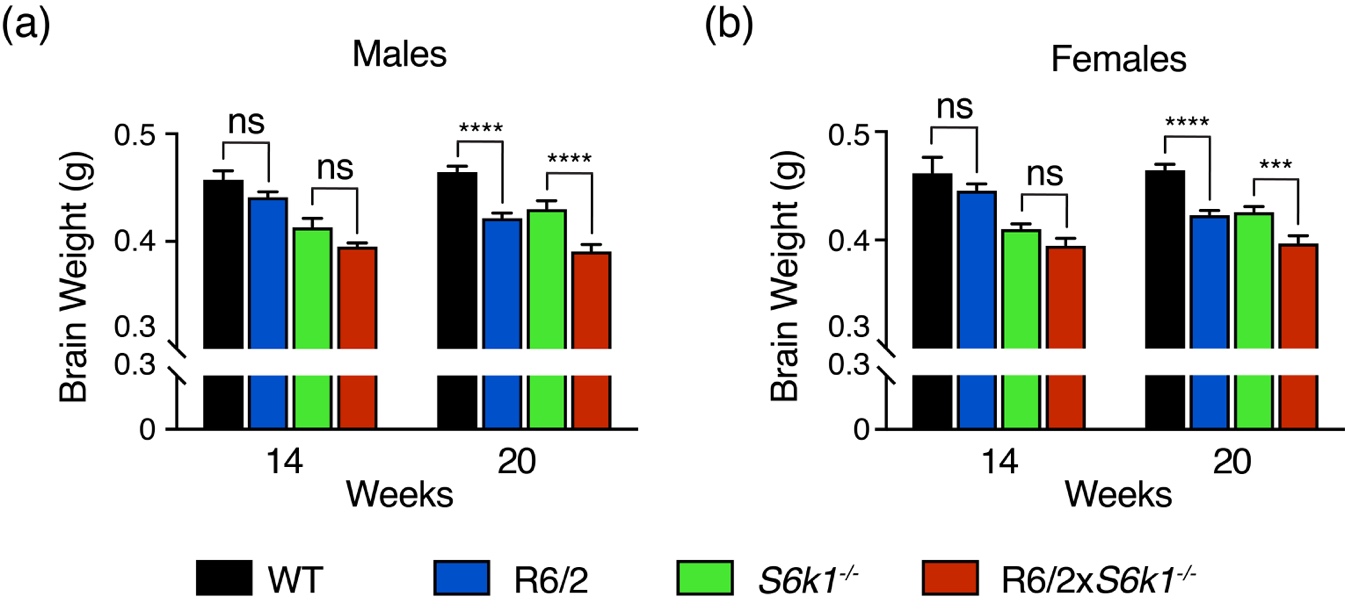
**

**Supplementary Figure 3: Brain weight is reduced in both R6/2 and R6/2x*S6k1^-/-^* mice.** Brain weights of male (a) and female (b) mice at 14 and 20 weeks of age. At 20 weeks of age both male and female R6/2 mice had significantly reduced brain weight compared to their WT littermates (P < 0.0001). For 14 weeks, males n = 4-9/genotype and females n = 4-5/genotype and for 20 weeks, males n = 12-14/genotype and females n = 14/genotype. Data were analysed by GLM univariate and ANOVA with Bonferroni correction. *** P < 0.001 and **** P < 0.0001.

**References**

1 Hunter, R. W. *et al.* Metformin reduces liver glucose production by inhibition of fructose-1-6-bisphosphatase. *Nature Medicine* **24**, 1395-1406, doi:10.1038/s41591-018-0159-7 (2018).

2 Maddala, R. *et al.* Rac1 GTPase-deficient mouse lens exhibits defects in shape, suture formation, fiber cell migration and survival. *Developmental Biology* **360**, 30-43, doi:<https://doi.org/10.1016/j.ydbio.2011.09.004> (2011).
